# Gastrointestinal toxicity associated with cyclin-dependent kinase 4/6 inhibitors in breast cancer patients: insights from a real-world pharmacovigilance analysis

**DOI:** 10.3389/fmed.2026.1876415

**Published:** 2026-07-15

**Authors:** Mengjiao Lu, Lei Yuan, Junjie Liu, Yifeng Dou, Jinguo Cui

**Affiliations:** 1 Department of Pharmacy, Tianjin Baodi Hospital, Tianjin Medical University Baodi Hospital, Tianjin, China; 2 Network Information Center, Tianjin Baodi Hospital, Tianjin Medical University Baodi Hospital, Tianjin, China

**Keywords:** breast cancer, cyclin-dependent kinase 4/6 inhibitors, FAERS, gastrointestinal toxicity, pharmacovigilance

## Abstract

**Background:**

Cyclin-dependent kinase 4/6 (CDK4/6) inhibitors play an important role in the treatment of breast cancer and show therapeutic potential for gastrointestinal (GI) tumors. However, their real-world GI safety profile remains incompletely characterized.

**Methods:**

Disproportionate reporting signals of GI adverse events (AEs) were assessed using the ROR, PRR and BCPNN algorithms, based on data from the FDA Adverse Event Reporting System database (2004-Q1 2025). Time-to-onset was analyzed using Weibull distribution, and serious vs. non-serious AE comparisons used non-parametric tests.

**Results:**

A total of 63,722 reports were associated with CDK4/6 inhibitors in breast cancer patients, among which 18,589 involved GI AEs. The three agents showed heterogeneous GI reporting patterns, with abdominal discomfort being the sole identified class-wide effect. Palbociclib accounted for the largest proportion of CDK4/6 inhibitor-related GI AE reports among the three agents, and its disproportionate reporting signals primarily involved oropharyngeal and upper GI events, with lip exfoliation and tongue blistering identified as its strongest signals. Ribociclib had the highest proportion of serious GI reports among the three drugs (82.38%), with 14.25% of its reports having a fatal outcome, and showed significant disproportionality signals for reflux gastritis and dysbiosis. For abemaciclib, gastrointestinal disorders was the most frequently reported System Organ Class, and diarrhea was its strongest disproportionate reporting signal. Notably, over 80% of positive GI pharmacovigilance signals detected in this study were absent from current prescribing information. All agents exhibited an early failure-type time-to-onset profile (β < 1), with a statistically significant difference in median onset time across agents: 15 days for abemaciclib, 27 days for ribociclib, and 49 days for palbociclib (*p* < 0.001). Serious GI AEs were more frequently reported in patients with advanced age, low weight, ribociclib exposure, and concomitant therapy with aromatase inhibitors or fulvestrant.

**Conclusion:**

This study revealed heterogeneous disproportionate reporting patterns for GI AEs among CDK4/6 inhibitors and identified unlabeled pharmacovigilance signals. These findings are exclusively hypothesis-generating pharmacovigilance observations, not evidence of incidence, causal associations, or clinically confirmed inter-drug risk differences. The results might help prioritize drug-event pairs for further research and require validation in well-designed observational or prospective studies.

## Introduction

Breast cancer has emerged as one of the most severe threats to women's health worldwide. According to the latest statistical data from the global cancer observatory (GLOBOCAN) released by the international agency for research on cancer (IARC) (1), breast cancer ranks second in incidence among all malignancies worldwide and fourth in mortality. Among the various subtypes of breast cancer, hormone receptor-positive (HR+), human epidermal growth factor receptor 2-negative (HER2-) accounts for up to 70% of cases (2). Treatment for these patients has long relied on endocrine therapy, but drug resistance often leads to treatment failure. The emergence of CDK4/6 inhibitors has broken this therapeutic impasse. By specifically inhibiting the activity of CDK4 and CDK6 kinases, CDK4/6 inhibitors block the phosphorylation of the retinoblastoma protein, arresting the cell cycle at the G1 phase and thereby effectively suppressing the abnormal proliferation of tumor cells (3). Currently, the CDK4/6 inhibitors approved for marketing by the U.S. food and drug administration (FDA) include palbociclib, ribociclib, and abemaciclib, all of which are indicated for locally advanced or metastatic breast cancer (4–6). Among them, ribociclib and abemaciclib have also been approved for early-stage breast cancer patients at high risk of recurrence (5, 6).

The expanding clinical use of CDK4/6 inhibitors has drawn increased attention to their associated safety profiles. Among various adverse events, gastrointestinal (GI) AEs are one of the most commonly reported toxic reactions, mainly including diarrhea, nausea, and vomiting (7–9). Diarrhea was reported as the key GI toxicity for abemaciclib (10). These reactions are of particular clinical concern as they frequently lead to dose reductions or treatment interruptions, potentially compromising antitumor efficacy and potentially impacting long-term survival. In addition, CDK4/6 inhibitors have shown potential value in gastrointestinal cancers, becoming a major research focus recently. Multiple studies have demonstrated that CDK4/6 inhibitors enhanced the sensitivity of gastric cancer, colorectal cancer, and other gastrointestinal malignancies to chemotherapy or immunotherapy (11–13). Consequently, a systematic evaluation of their GI safety of CDK4/6 inhibitors in breast cancer patients is critical, not only for optimizing the treatment management in breast cancer but also for offering critical toxicological references for their future application in gastrointestinal tumors.

A meta-analysis by Shohdy et al. (14), which pooled four randomized phase II/III trials (*n* = 2,007), established diarrhea, nausea, vomiting, and decreased appetite as the principal GI AEs associated with CDK4/6 inhibitors, aligning with drug labels. However, the applicability of these results to real-world populations remains limited due to the common exclusion of vulnerable subgroups, such as elderly patients and those with significant comorbidities, from clinical trials. It is necessary to conduct a large-scale real-world study to systematically evaluate the GI toxicity, temporal dynamics, and risk factors of different CDK4/6 inhibitors. The FDA Adverse Event Reporting System (FAERS) database provides a valuable resource for this purpose, offering a large and diverse dataset for identifying potential safety signals that may not be evident in controlled clinical trials. However, as Khouri et al. (15) warned, the misuse of spontaneous reporting systems to draw causal conclusions or make comparative risk assessments poses a significant threat to evidence-based medicine. This study therefore leveraged the FAERS database to detect and characterize potential differences in GI toxicity profiles among CDK4/6 inhibitors, aiming to generate hypotheses for targeted monitoring and future etiological or observational research.

## Materials and methods

### Data source

This study utilized data from the FAERS database, a comprehensive and widely recognized pharmacovigilance database that aggregates spontaneous safety reports for marketed pharmaceutical and biologic products. To ensure both historical depth and contemporary relevance, data spanning from the first quarter of 2004 through the first quarter of 2025 were downloaded in ASCII format from the official FAERS public dashboard (https://fis.fda.gov/extensions/FPD-QDE-FAERS/FPD-QDE-FAERS.html). For each quarter, seven distinct data files were retrieved: patient demographic and administrative information (DEMO), drug/biologic exposure data (DRUG), adverse event terms (REAC), patient outcomes (OUTC), report sources (RPSR), drug therapy start and end dates (THER), and indications for use (INDI). All analyses were performed using SAS software (version 9.4), which is explicitly recommended by the FDA for the mining of this database. Ethical approval was not required for this study, as the analysis was based on publicly available, de-identified data from the FAERS.

### Data processing

A structured, multi-stage data processing workflow was implemented in this study to transform the raw FAERS data into a refined and analyzable dataset. De-duplication algorithm recommended by FDA was applied to address duplicate case reports. Specifically, for each unique CASEID, the report with the most recent FDA_DT (FDA receipt date) was retained. In cases where multiple reports shared identical receipt dates, the report with the highest PRIMARYID was selected to ensure data uniqueness. Drug nomenclature was standardized to their generic active ingredient names to consolidate trade names and synonyms, and only reports where CDK4/6 inhibitors were labeled as the primary suspected drug (PS) were included. AEs were systematically coded, classified, and described using the preferred terms (PTs) and system organ classes (SOCs) of the medical dictionary for regulatory activities (MedDRA version 28.0). Gastrointestinal disorders were specifically identified under SOC code 10017947 for subsequent analysis.

### Signal mining

Disproportionality analysis serves as a fundamental hypothesis-generating tool for identifying potential associations between specific drugs and AEs, while providing a statistical foundation for subsequent clinical assessment of case reports. It does not establish causality, incidence, or comparative risk. In this study, three well-established methodologies were employed for AEs signal detection: the Reporting Odds Ratio (ROR) (16), the Proportional Reporting Ratio (PRR) (17), and the Bayesian confidence propagation neural network (BCPNN) (18). At the SOC level, the ROR method was used to assess associations between drugs and AEs. At the PT level, a conservative signaling strategy was adopted: an AE signal was considered statistically significant only when it simultaneously met the detection criteria of all three algorithms. The calculation formulas and signaling thresholds for each method followed internationally recognized pharmacovigilance guidelines and previous methodological literature, as detailed in Supplementary Table S1. As emphasized by Khouri et al. (15) and Fusaroli et al. (19), all disproportionality signals identified herein indicate only that certain drug-event pairs were reported more frequently than expected statistically, and do not imply a causal relationship between the drug and the event. A positive disproportionality signal for an AE not listed in the FDA label was classified as a potential unlabeled reporting signal, rather than a confirmed novel adverse drug reaction.

### Time-to-onset analysis

The time-to-onset (TTO) of GI AEs following CDK4/6 inhibitor initiation was analyzed using the reported event dates from the FAERS database. The onset time for each case was calculated as the interval between the start date of the suspect drug (START_DT) and the date the adverse event occurred (EVENT_DT). Only cases with complete and valid date information were included in this analysis. Weibull shape parameter (WSP) testing was employed to evaluate the hazard profile of the adverse events over time. To further summarize the central tendency and dispersion of the onset distribution, the median TTO with interquartile range (IQR) was computed.

### Statistical analysis

All statistical analyses were performed using SAS software (version 9.4), IBM SPSS Statistics (Version 28.0) and Microsoft Excel 2021.

## Results

### Descriptive analysis

**Figure 1:**
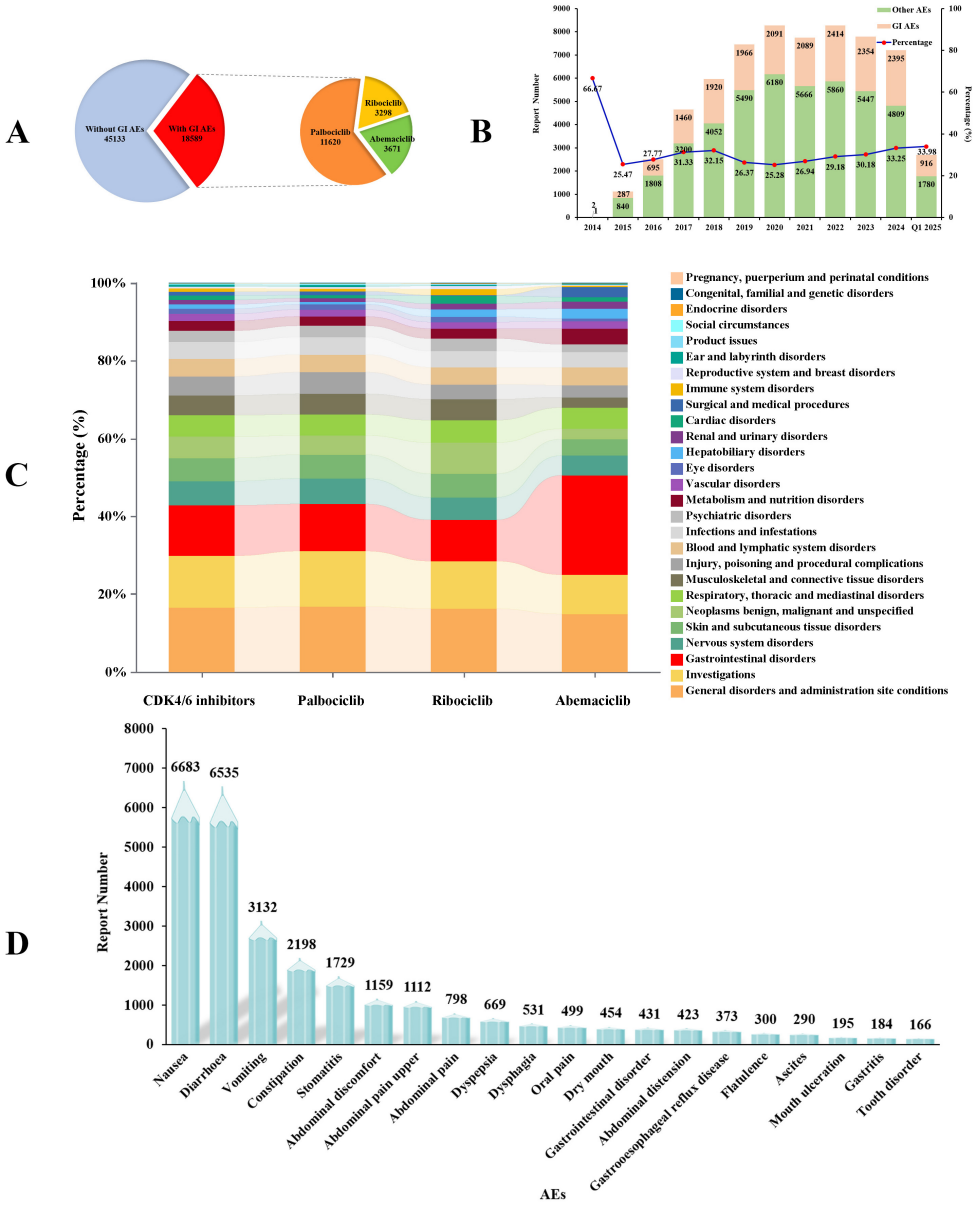
Descriptive analysis of GI AEs of CDK4/6 inhibitors from the FAERS database between 2004 and Q1 2025. **(A)** Distribution of GI AEs among CDK4/6 inhibitors. **(B)** Annual number and proportion of CDK4/6 inhibitor-associated GI AE reports. **(C)** Distribution of CDK4/6 inhibitor-associated AEs in SOCs. **(D)** Top 20 most frequently reported CDK4/6 inhibitor-associated GI AEs.

Between 2004 and Q1 2025, the FAERS database contained 63,722 reports of CDK4/6 inhibitors in breast cancer patients (254,521 adverse event occurrences). Of these, 18,589 reports documented GI events, accounting for 32,942 GI adverse event occurrences. Palbociclib contributed the majority of reports, followed by abemaciclib and ribociclib (Figure 1A). Over the study period, the annual proportion of GI AEs increased from 25.47% to 33.98%, peaking in 2018, reaching a trough in 2020, and and then rising steadily thereafter (Figure 1B). At the SOC level, GI AEs represented the highest proportion (25.53%) among all 27 SOC categories for abemaciclib, whereas for palbociclib, ribociclib, and the overall cohort, GI AEs ranked third, with proportions of 12.10%, 10.58%, and 12.94%, respectively (Figure 1C). In terms of specific PTs (Figure 1D), the most frequently reported GI AEs overall were nausea (*n* = 6,683) and diarrhea (*n* = 6,535), followed by vomiting (*n* = 3,132), constipation (*n* = 2,198), and stomatitis (*n* = 1,729).

Clinical characteristics of GI AEs associated with CDK4/6 inhibitors are summarized in Table 1. A pronounced sex-based disparity was observed, with AEs reported predominantly in female patients (97.70%) compared to male patients (0.90%), along with 260 cases (1.40%) of unspecified sex. This distribution aligns with the primary indication of CDK4/6 inhibitors for the treatment of breast cancer, which occurs largely in women. Patients aged ≥65 years represented the largest age group (40.48%), particularly in the palbociclib group (50.54%). The median age across the overall cohort was 64 years, with that of palbociclib being slightly older (65 years) compared to those of ribociclib (61 years) and abemaciclib (61 years). Consumers submitted the majority of AE reports (52.52%), followed by pharmacists (22.55%). Geographically, the United States contributed more than two-thirds of all reports (72.13%), especially for palbociclib (83.80%). In this database, serious reports accounted for 54.12% of all reports, with the highest proportion in the ribociclib group (82.38%). The most common serious outcomes were other serious outcomes (40.67%), hospitalization (21.66%), and death (6.57%). Ribociclib showed notably higher proportions of life-threatening event reports (6.43%) and death reports (14.25%) compared to other agents in this database. Approximately half of the reports (51.11%) involved concomitant medications, with the highest reporting rate in the palbociclib group (56.33%) and the highest rate of monotherapy reports (70.25%) for abemaciclib.

**Table 1:** Demographic characteristics of breast cancer patients with CDK4/6 inhibitor-associated gastrointestinal adverse event reports.

Characteristics, *n* (%)	All CDK4/6 inhibitors (*n* = 18,589)	Palbociclib (*n* = 11,620)	Ribociclib (*n* = 3,298)	Abemaciclib (*n* = 3,671)
Gender				
Female	18,161 (97.70)	11,444 (98.49)	3,231 (97.97)	3,486 (94.96)
Male	168 (0.90)	111 (0.96)	20 (0.61)	37 (1.01)
Not specified	260 (1.40)	65 (0.56)	47 (1.43)	148 (4.03)
Age				
< 18	1 (0.01)	1 (0.01)	0 (0.00)	0 (0.00)
18–44	1,054 (5.67)	563 (4.85)	246 (7.46)	245 (6.67)
45–64	6,800 (36.58)	4,683 (40.30)	883 (26.77)	1,234 (33.61)
≥65	7,525 (40.48)	5,873 (50.54)	760 (23.04)	892 (24.30)
Not specified	3,209 (17.26)	500 (4.30)	1,409 (42.72)	1,300 (35.41)
Mean ± SD	63.64 ± 12.21	64.87 ± 11.80	60.25 ± 13.19	60.57 ± 12.27
Median (IQR)	64 (56–72)	65 (57–73)	61 (50–70)	61 (52–69)
Reporter				
Consumer	9,763 (52.52)	5,128 (44.13)	2,106 (63.86)	2,529 (68.89)
Pharmacist	4,192 (22.55)	3,224 (27.75)	415 (12.58)	553 (15.06)
Physician	2,061 (11.09)	1,062 (9.14)	652 (19.77)	347 (9.45)
Other health-professional	2,339 (12.58)	2,087 (17.96)	86 (2.61)	166 (4.52)
Not specified	234(1.26)	119 (1.02)	39 (1.18)	76 (2.07)
Reporting country (Top 3)				
1	USA 13,408 (72.13)	USA 9,738 (83.80)	USA 833 (25.26)	USA 2,837 (77.28)
2	India 563 (3.03)	India 426 (3.67)	Brazil 315 (9.55)	Japan 167 (4.55)
3	Argentina 403 (2.17)	Argentina 262 (2.25)	Germany 277 (8.40)	China 82 (2.23)
Serious report				
Serious	10,061 (54.12)	6,080 (52.32)	2,717 (82.38)	1,264 (34.43)
Non-Serious	8,528 (45.88)	5,540 (47.68)	581 (17.62)	2,407 (65.57)
Outcome^*^				
Life–threatening	310 (1.67)	75 (0.65)	212 (6.43)	23 (0.63)
Hospitalization initial or prolonged	4,026 (21.66)	2,215 (19.06)	1,146 (34.75)	665 (18.11)
Disability	111 (0.60)	38 (0.33)	57 (1.73)	16 (0.44)
Death	1,221 (6.57)	666 (5.73)	470 (14.25)	85 (2.32)
Congenital anomaly	2 (0.01)	0 (0.00)	1 (0.03)	1 (0.03)
Required intervention to prevent permanent impairment/damage	22 (0.12)	12 (0.10)	5 (0.15)	5 (0.14)
Other outcomes	7,560 (40.67)	4,755 (40.92)	2,093 (63.46)	712 (19.40)
Concomitant medications				
Monotherapy	9,088 (48.89)	5075 (43.67)	1424 (43.18)	2579 (70.25)
Concomitant medications	9,501 (51.11)	6545 (56.33)	1,874 (56.82)	1,092 (29.75)

IQR, interquartile range; SD, standard deviation. ^*^The patients can have multiple serious adverse event outcomes.

### Disproportionality analysis

**Figure 2:**
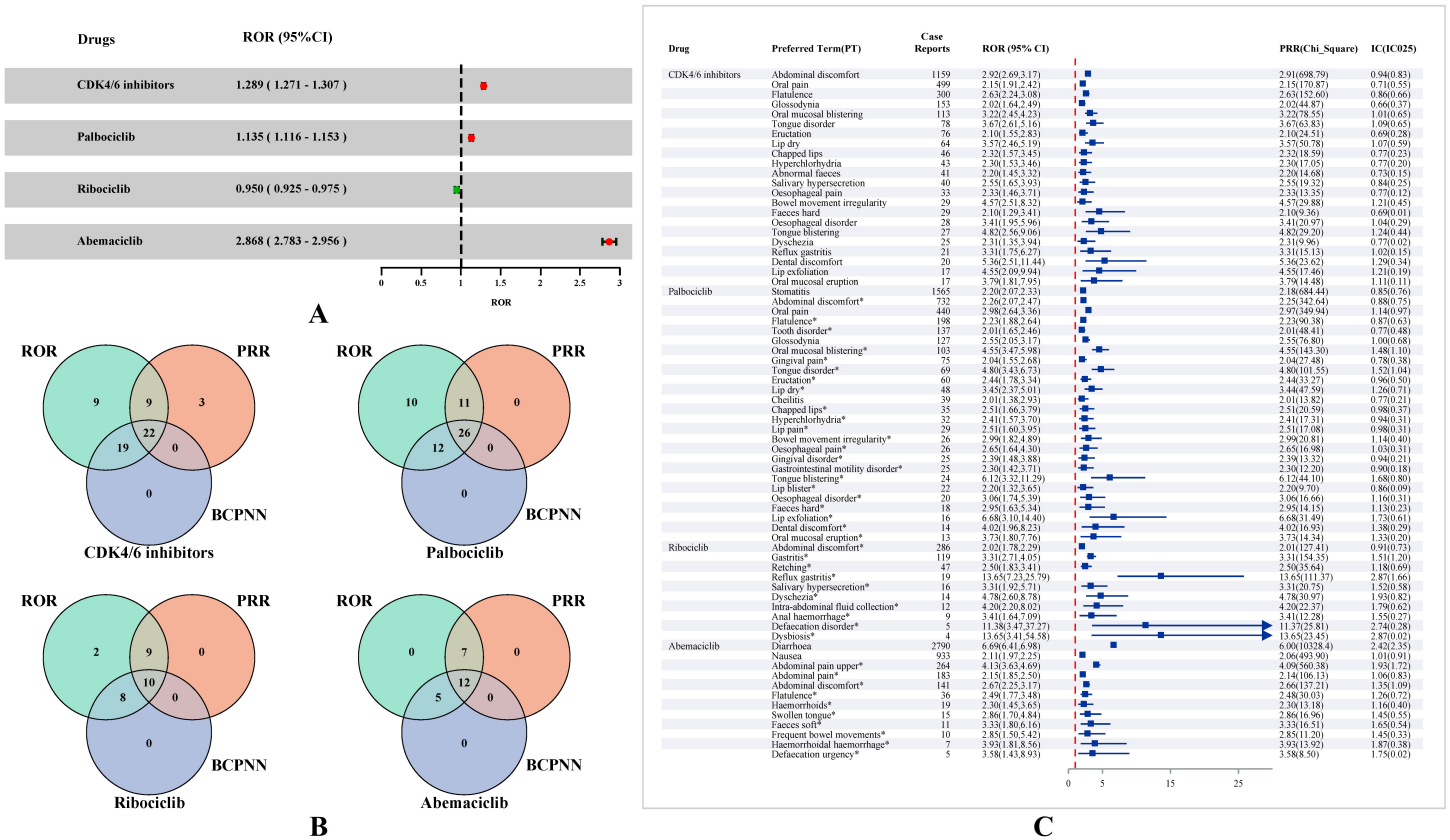
Statistical association between GI AEs and the use of CDK 4/6 inhibitors. **(A)** Forest plot of ROR for the SOC of gastrointestinal disorders associated with CDK4/6 inhibitors. **(B)** Venn diagram of PT-level disproportionality signals meeting the criteria of three algorithms. **(C)** Forest plot of disproportionality analysis for GI AEs at the PT level. *Adverse event has not been mentioned in the prescribing information.

**Figure 3:**
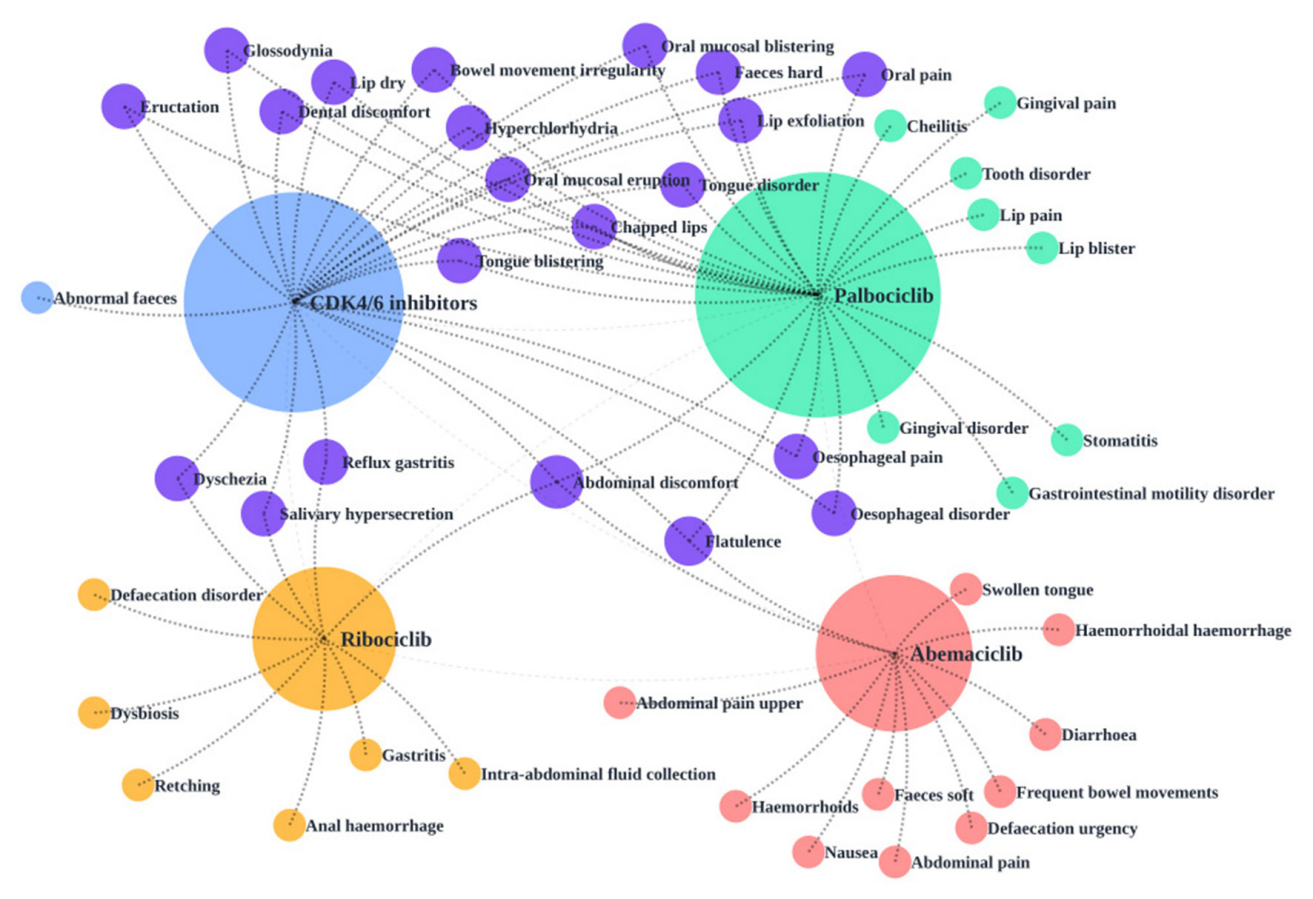
Network plot of GI disproportionality signals associated with CDK4/6 inhibitors. Central nodes represent the overall drug class (blue) and individual agents: palbociclib (green), ribociclib (orange), and abemaciclib (red). Surrounding peripheral nodes denote individual AE terms, with node size proportional to the number of connected drugs. Dashed lines indicate statistically significant associations between a drug and a specific AE.

**Figure 4:**
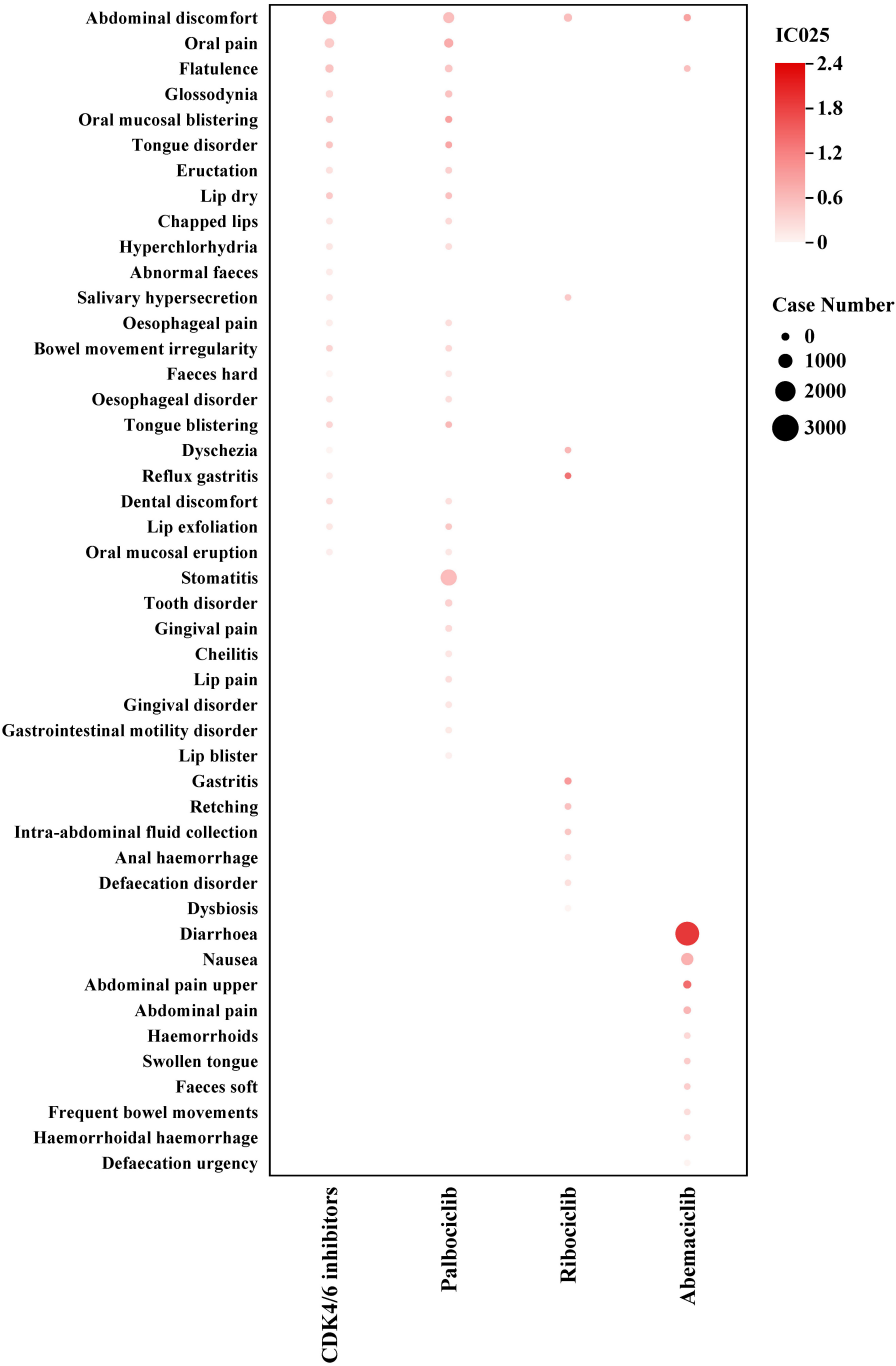
Heatmap of CDK4/6 inhibitor-associated GI AEs based on IC025 and reporting number.

GI AE disproportionality signals associated with CDK4/6 inhibitors were analyzed using the ROR, PRR, and BCPNN methods. At the SOC level, disproportionality analysis (Figure 2A) revealed a positive signal for the CDK4/6 inhibitor class (ROR = 1.29). Among individual agents, abemaciclib showed the strongest association (ROR = 2.87), followed by palbociclib (ROR = 1.13), whereas ribociclib was not significantly associated (ROR = 0.95). At the PT level, a total of 22 signals were identified for the CDK4/6 inhibitor class, with 26 signals for palbociclib, 12 for abemaciclib, and 10 for ribociclib (Figure 2B). As detailed in Figure 2C, in the overall cohort, abdominal discomfort was the most frequently reported PT for the overall cohort (*n* = 1,159) and generated a significant signal (ROR = 2.92). Other notable signals included oral pain, flatulence, and several oral mucosal events. Strong associations were also observed for less common events such as tongue blistering, dental discomfort, and bowel movement irregularity. For palbociclib, strong signals were identified for stomatitis (*n* = 1,565, ROR = 2.20), abdominal discomfort (*n* = 732, ROR = 2.26), and oral pain (*n* = 440, ROR = 2.98), with notably high association strengths observed for lip desquamation (ROR = 6.68), tongue blistering (ROR = 6.12), and tongue disorders (ROR = 4.80). Ribociclib was associated with a pronounced signal for reflux gastritis (ROR = 13.65), which was notably stronger than that for general gastritis (ROR = 3.31). Similarly strong signals were observed for defaecation disorder (ROR = 11.38) and dysbiosis (ROR = 13.65), despite low case counts (*n* = 5 and *n* = 4, respectively). Abemaciclib was most strongly associated with diarrhea (*n* = 2,790, ROR = 6.69), which was both the most frequent and the highest-strength signal. Meanwhile, significant signals including upper abdominal pain (ROR = 4.13), haemorrhoidal hemorrhage (ROR = 3.93), and defaecation urgency (ROR = 3.58) were also detected. Notably, the majority of these positive PT-level signals were absent from the corresponding drug labels, with all ribociclib signals, 10 of 12 abemaciclib signals, and 22 of 26 palbociclib signals being unlabeled (Figure 2C). In addition, among all positive signals, only abdominal discomfort was shared by all four cohorts (Figure 3), with signal strength ranked as follows: overall CDK4/6 inhibitor cohort > abemaciclib > palbociclib > ribociclib. A heatmap utilizing IC025 values (Figure 4) was constructed to provide an enhanced visual representation of these findings.

### Early-onset patterns and weibull analysis

**Figure 5:**
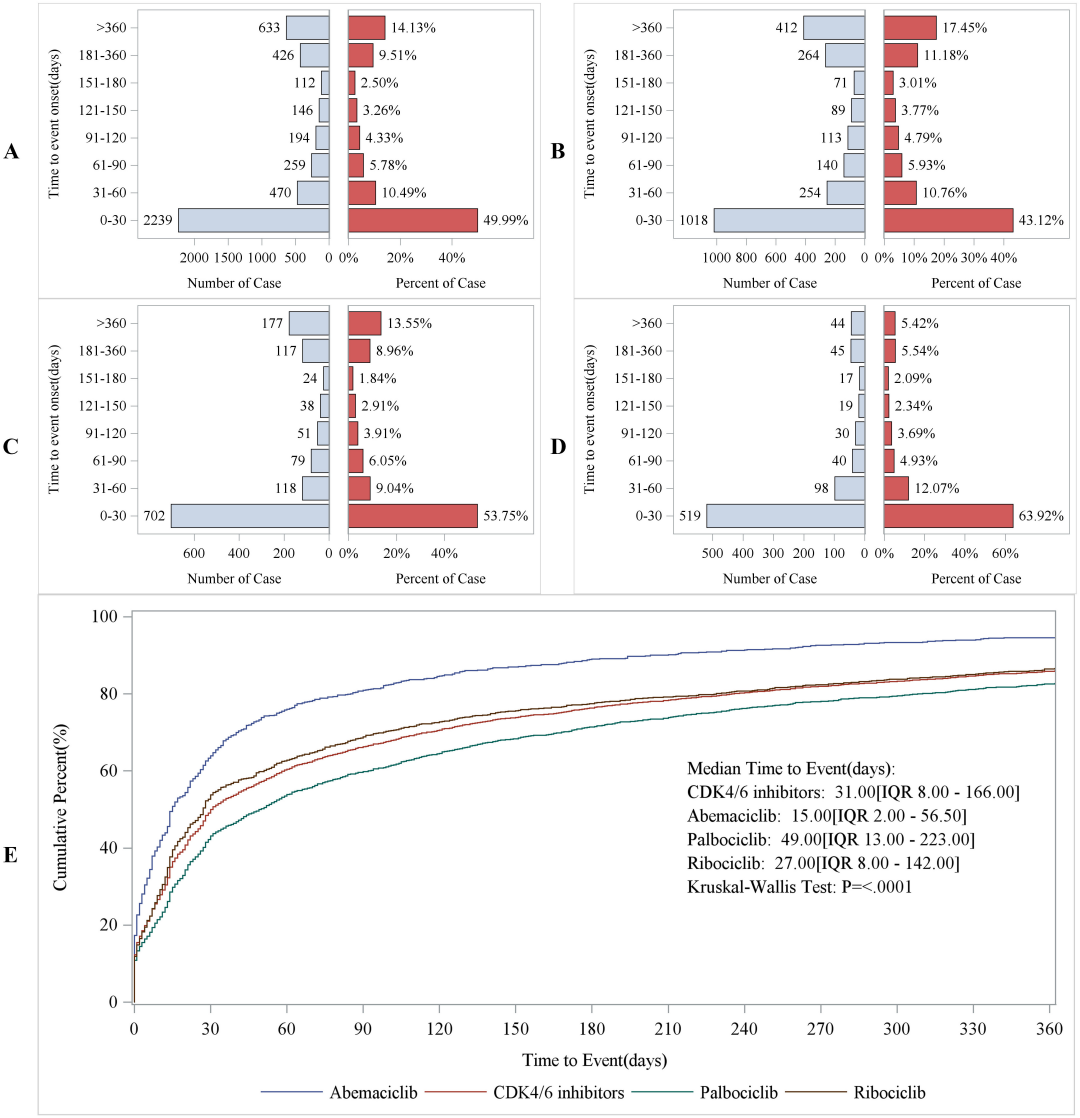
Time to onset of CDK4/6 inhibitor-associated GI AEs. **(A-D)** Time to onset distribution of GI AEs reports for the overall CDK4/6 inhibitors, palbociclib, ribociclib and abemaciclib, respectively. **(E)** Cumulative proportion of reported GI AEs by time. Statistical testing was performed using the nonparametric Kruskal–Wallis test.

Table 2 and Figure 5 summarized the TTO and Weibull modeling results for CDK4/6 inhibitor-associated GI AEs. A total of 4,479 valid TTO cases were identified, including 2,361 for palbociclib, 1,306 for ribociclib, and 812 for abemaciclib. As shown in Figures 5A–D, nearly half of all GI events occurred within the first 30 days, with notable inter-drug differences in TTO profiles. Abemaciclib exhibited the highest early-onset percentage, with 63.92% of cases occurring within 0–30 days, followed by a sharp decline. Ribociclib (53.75%) and palbociclib (43.12%) also showed high early incidence, but their decline over the subsequent 180 days was more gradual. Moreover, a substantial proportion of palbociclib (17.45%) and ribociclib (13.55%) cases were reported after 360 days, a pattern not observed for abemaciclib (5.42%). These temporal patterns were reflected in the median TTO: abemaciclib had the shortest median TTO of 15.0 days (IQR: 2.0–56.5 days), followed by ribociclib (27.0 days, IQR: 8.0–142.0 days), and palbociclib (49.0 days, IQR: 13.0–223.0 days). The cumulative percentage of GI AEs differed significantly among the three agents (Kruskal–Wallis test, *p* < 0.05; Figure 5E).

**Table 2:** Weibull distribution analysis for the time to onset of reported CDK4/6 inhibitors-associated gastrointestinal adverse events.

Drug	Case reports	Scale parameter: α (95%CI)	Shape parameter: β (95%CI)	Type
All GI AEs				
CDK4/6 inhibitors	4,479	122.14 (115.62–129.02)	0.60 (0.59–0.62)	Early failure
Palbociclib	2,361	159.34 (148.30–171.19)	0.63 (0.61–0.65)	Early failure
Ribociclib	1,306	109.06 (98.48–120.77)	0.60 (0.57–0.63)	Early failure
Abemaciclib	812	57.26 (50.25–65.24)	0.62 (0.58–0.65)	Early failure
Abdominal discomfort				
CDK4/6 inhibitors	246	140.69 (112.19–176.43)	0.64 (0.58–0.71)	Early failure
Palbociclib	121	140.11 (105.65–185.81)	0.71 (0.61–0.81)	Early failure
Ribociclib	93	183.77 (123.28–273.92)	0.60 (0.50– 0.71)	Early failure
Abemaciclib	32	47.22 (25.90–86.08)	0.72 (0.54–0.96)	Early failure

CI, confidence interval.

Weibull distribution analysis confirmed an early-failure pattern for all CDK4/6 inhibitors (Table 2), with shape parameters (β) significantly below 1 (range: 0.60–0.72), indicating decreasing hazard rates over time. The scale parameter (α) for overall GI AEs was longest for palbociclib (159.34 days), approximately three times that of abemaciclib (57.26 days), with ribociclib falling in between (109.06 days). This pattern persisted for abdominal discomfort, the only AE common to all three drugs, where abemaciclib again showed the shortest α value (47.22 days), further supporting its distinct rapid-onset profile.

### Differential features of serious and non-serious adverse events

**Figure 6:**
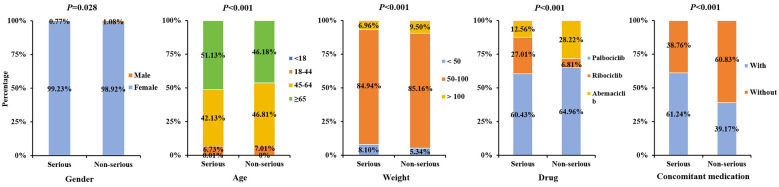
Characteristics of serious and non-serious GI AEs reports associated with CDK4/6 inhibitors.

A comprehensive analysis of 18,589 adverse event cases revealed significant differences in characteristics between serious (*n* = 10,061) and non-serious (*n* = 8,528) cases, as detailed in Table 3 and Figure 6. Demographically, the serious case group had a higher proportion of female patients (98.02 vs. 97.31%, *p* = 0.028) and was characterized by an older patient population with a higher median age (65 years vs. 63 years, *p* < 0.001). Weight distribution patterns further distinguished the groups, with serious cases showing a lower median weight (68.48 kg vs. 72.11 kg, *p* < 0.001) and a higher proportion of patients in the lower weight categories. Marked differences also emerged in drug distribution patterns between serious and non-serious cases. Ribociclib was disproportionately associated with serious cases (27.01 vs. 6.81%, *p* < 0.001), whereas palbociclib (60.43 vs. 64.96%, *p* < 0.001) and abemaciclib (12.56 vs. 28.22%, *p* < 0.001) both were more common in non-serious reports. Concomitant medication use was substantially higher in serious cases (61.24 vs. 39.17%, *p* < 0.001). This pattern was particularly evident for aromatase inhibitors (38.02 vs. 22.43%, *p* < 0.001) and fulvestrant (18.12 vs. 9.38%, *p* < 0.001). In contrast, CYP3A4 inhibitor usage showed no significant difference between groups (2.07 vs. 1.95%, *p* = 0.559). Regarding specific adverse events, serious cases demonstrated significantly higher reporting rates for several GI events, including dyschezia (0.24 vs. 0.01%), reflux gastritis (0.19 vs. 0.02%), salivary hypersecretion (0.32 vs. 0.09%), abnormal feces (0.32 vs. 0.11%,), hyperchlorhydria (0.33 vs. 0.12%), and eructation (0.53 vs. 0.27%) (all *p* < 0.01). Conversely, oral pain, tongue disorder, oesophageal pain, and oesophageal disorder were more frequently reported in non-serious cases.

**Table 3:** Differences in characteristics between serious and non-serious gastrointestinal adverse event reports.

Clinical characteristics, *n* (%)	Serious cases (*n* = 10,061)	Non-serious cases (*n* = 8,528)	Statistic	*p*-value
**Gender**				
Female	9,862 (98.02)	8,299 (97.31)	4.811^b^	0.028^a^
Male	77 (0.77)	91 (1.07)		
**Age**			−5.905^d^	< 0.001^c^
< 18	1 (0.01)	0 (0.00)		
18–44	574 (5.71)	480 (5.63)		
45–64	3,596 (35.74)	3,204 (37.57)		
≥65	4,364 (43.38)	3,161 (37.07)		
Not specified	1,526 (15.17)	1,683 (19.73)		
Median (IQR)	65 (56–73)	63 (55–72)		
**Weight (kg)**			−6.992^d^	< 0.001^c^
< 50	271 (2.69)	104 (1.22)		
50–100	2,843 (28.26)	1,659 (19.45)		
>100	233 (2.32)	185 (2.17)		
Not specified	6,714 (66.73)	6,580 (77.16)		
Median (IQR)	68.48 (58.96–81)	72.11 (61.22–85.63)		
**Drugs**			1,649.184^b^	< 0.001^a^
Palbociclib	6,080 (60.43)	5,540 (64.96)	40.437^b^	< 0.001^a^
Ribociclib	2,717 (27.01)	581 (6.81)	1,289.539^b^	< 0.001^a^
Abemaciclib	1,264 (12.56)	2,407 (28.22)	71.341^b^	< 0.001^a^
**Concomitant medications**				
Concomitant medications	6,161 (61.24)	3,340 (39.17)	899.839^b^	< 0.001^a^
Aromatase inhibitors^*^	3,825 (38.02)	1,913 (22.43)	525.438^b^	< 0.001^a^
Fulvestrant	1,823 (18.12)	800 (9.38)	290.827^b^	< 0.001^a^
CYP3A4 inhibitors^*^	208 (2.07)	166 (1.95)	0.342^b^	0.559^a^
**Types of AEs**				
Abdominal discomfort	655 (6.51)	504 (5.91)	2.846^b^	0.092^a^
Oral pain	225 (2.24)	274 (3.21)	16.851^b^	< 0.001^a^
Flatulence	179 (1.78)	121 (1.42)	3.773^b^	0.052^a^
Glossodynia	77 (0.77)	76 (0.89)	0.896^b^	0.344^a^
Oral mucosal blistering	52 (0.52)	61 (0.72)	3.008^b^	0.083^a^
Tongue disorder	32 (0.32)	46 (0.54)	5.412^b^	0.02^a^
Eructation	53 (0.53)	23 (0.27)	7.492^b^	0.006^a^
Lip dry	38 (0.38)	26 (0.30)	0.713^b^	0.398^a^
Chapped lips	23 (0.23)	23 (0.27)	0.316^b^	0.574^a^
Hyperchlorhydria	33 (0.33)	10 (0.12)	8.882^b^	0.003^a^
Abnormal feces	32 (0.32)	9 (0.11)	9.473^b^	0.002^a^
Salivary hypersecretion	32 (0.32)	8 (0.09)	10.810^b^	0.001^a^
Oesophageal pain	24 (0.24)	9 (0.11)	4.608^b^	0.032^a^
Feces hard	17 (0.17)	12 (0.14)	0.237^b^	0.627^a^
Bowel movement irregularity	17 (0.17)	12 (0.14)	0.237^b^	0.627^a^
Oesophageal disorder	21 (0.21)	7 (0.08)	4.922^b^	0.027^a^
Tongue blistering	18 (0.18)	9 (0.11)	1.713^b^	0.191^a^
Dyschezia	24 (0.24)	1 (0.01)	17.680^b^	< 0.001^a^
Reflux gastritis	19 (0.19)	2 (0.02)	11.189^b^	0.001^a^
Dental discomfort	12 (0.12)	8 (0.09)	0.278^b^	0.598^a^
Lip exfoliation	9 (0.09)	8 (0.09)	0.010^b^	0.992^a^
Oral mucosal eruption	11 (0.11)	6 (0.07)	0.767^b^	0.381^a^

IQR, interquartile range; CYP3A4, Cytochrome P450 Family 3 Subfamily A Polypeptide 4. The AEs listed above were AEs with significant signal strengths. A *p*-value < 0.05 was considered statistically significant. ^a^Proportions were compared using the Pearson Chi–squared test. ^b^χ^2^ statistic of the Pearson Chi–squared test. ^c^Mann–Whitney U test. ^d^Z statistic of the Mann–Whitney U test. ^*^Aromatase inhibitors include letrozole, anastrozole and exemestane. ^*^CYP3A4 inhibitors were systematically classified according to FDA system (45).

## Discussion

CDK4/6 inhibitors have emerged as the standard first-line therapeutic regimen for HR+/HER2- breast cancer, with well-documented efficacy in significantly improving patient prognosis. However, GI AEs, a prevalent non-hematological toxicity of these agents, can reduce treatment adherence, impair quality of life, and even result in treatment discontinuation (20–22). Distinct GI toxicity profiles have been observed among CDK4/6 inhibitors, yet systematic pharmacovigilance analyses based on large-scale real-world data remain relatively limited. In this hypothesis-generating study, leveraging data from the FAERS database (2004-Q1 2025), we performed a comprehensive analysis of CDK4/6 inhibitor-related GI AEs in breast cancer, and delineated the event spectrum, disproportionality signal strength, time-to-onset characteristics, and features of serious event reports, The identified disproportionalities require further validation to establish any causal relationships

A total of 63,722 adverse event reports related to CDK4/6 inhibitors were included from the FAERS database in this study, of which 18,589 reports involved GI events, accounting for 29.2% of the total reports. This proportion exhibited a fluctuating upward trend over the observation period, reaching 33.98% by the end of the study. At the SOC level, GI AEs accounted for the highest proportion among abemaciclib-related AEs (25.53%) and ranked third among all organ system-based AE categories for both palbociclib and ribociclib. These reporting patterns are directionally aligned with the GI tolerability profiles observed in pivotal clinical trials from the PALOMA, MONARCH, and MONALEESA series (23–27), while providing additional real-world granularity at the PT level. The MONARCH-2 (23) and MONARCH-3 (24) trials demonstrated that the incidence of diarrhea with abemaciclib reached 80%−90%, with the incidence of grade ≥3 diarrhea reaching 10%−15%, representing the most predominant non-hematological toxicity of the agent. In contrast, the PALOMA trial series (25, 26) showed that the incidence of GI AEs of palbociclib was significantly lower than that of abemaciclib, and the MONALEESA trial (27) indicated an overall low level of GI toxicity for ribociclib. In the present study, GI AEs accounted for one-quarter of total abemaciclib-related AEs, a pattern that aligns with the recognized GI tolerability profile of abemaciclib (23, 24). One potential mechanistic hypothesis for this observed reporting pattern relates to its biochemical property of higher inhibitory selectivity for CDK4 over CDK6 (28, 29). CDK4 is expressed in normal human intestinal epithelium and is tightly linked to cell proliferation activity (30). Thus, highly selective inhibition of CDK4 could potentially contribute to intestinal epithelial injury and secretory dysfunction. Whether this reporting pattern relates to abemaciclib's greater CDK4 selectivity remains speculative and cannot be determined from these data. Moreover, the progressive increase in the proportion of GI AEs observed over time in the real-world setting may reflect both heightened awareness and reporting of GI toxicity among clinicians and patients, as well as the cumulative effect of an expanding exposed population driven by the broader clinical adoption of CDK4/6 inhibitors. These reports exhibited consistent demographic features that are predominantly female and concentrated in the over-45 age group, a pattern that aligns precisely with the well-established epidemiology of breast cancer.

A systematic disproportionality signal detection of GI AEs associated with CDK4/6 inhibitors was performed using three well-established pharmacovigilance algorithms: ROR, PRR, BCPNN. At the SOC level, the overall cohort generated a positive signal, indicating a statistical association between these agents and GI toxicity. Abemaciclib exhibited the strongest association, followed by palbociclib, whereas ribociclib did not meet the significance threshold. In the in-depth analysis at the PT level, multiple positive disproportionate reporting signals were identified for each of the three individual agents and the overall cohort. Of these, abdominal discomfort was the only positive disproportionality signal shared by all four cohorts, suggesting a class effect of CDK4/6 inhibitors on intestinal smooth muscle function or visceral sensitivity rather than a drug-specific toxicity phenotype. The three agents displayed markedly heterogeneous reporting patterns in terms of target organ preference, disproportionality signal strength, and proportion of serious event reports. This comparison, however, reflects only the reporting pattern differences observed in this database and should not be viewed as establishing comparative safety or causality, as they may be influenced by multiple factors including reporting bias, channeling bias, differences in drug utilization patterns, and variations in clinical practice across regions (15, 19).

Palbociclib exhibited a disproportionality signal profile most prominently characterized by oral mucosal events, with concurrent signals of lower GI AEs. The detection of a series of signals, such as stomatitis (ROR = 2.20), oral pain (ROR = 2.98), lip exfoliation (ROR = 6.68), tongue blistering (ROR = 6.12), and tooth disorder (ROR = 2.01) raised the hypothesis of potential effects of palbociclib on different anatomical regions of the oral cavity that require further investigation. The prescribing information for palbociclib defines stomatitis as encompassing aphthous oral mucositis, cheilitis, glossitis, glossodynia, and oral ulcers, whereas PT signals including lip desquamation and tongue blistering identified in this study are not listed as independent entries therein (4), suggesting that the oral adverse event reporting spectrum in the real-world setting may be more detailed and extensive than that described in the prescribing information. The mechanisms underlying the oral toxicities of palbociclib have not been fully elucidated, and dedicated mechanistic studies are currently lacking. Previous investigations have demonstrated that palbociclib can markedly suppress the proliferation, migration, and invasive capacity of oral squamous cell carcinoma cells through direct cell cycle inhibition, induction of DNA damage, and promotion of cellular senescence and apoptosis (31, 32). However, whether palbociclib exerts its oral toxicity through these very mechanisms remains to be further investigated. Furthermore, we hypothesized that the known immunosuppressive effect of palbociclib, particularly neutropenia, may impair local immune function within the oral cavity, thereby exacerbating mucosal injury (33, 34). Beyond its oral mucosal effects, palbociclib also generated positive lower GI signals, such as abdominal discomfort and bowel movement irregularity, although both the frequency and signal strength were considerably lower than those observed for oral mucosal events. Blume et al. demonstrated that in a murine intestinal organoid model, pretreatment with palbociclib transiently arrested intestinal epithelial cells in the G1 phase, thereby protecting intestinal organoids against the cytotoxicity of gemcitabine and SN-38, the cytotoxic metabolite of irinotecan (33). Xiang et al. (35) further revealed that palbociclib accelerated the recovery of chemotherapy-induced intestinal damage by protecting active intestinal stem cells and activating quiescent intestinal stem cells, without compromising the antitumor efficacy of cytotoxic chemotherapy on tumor grafts. Notably, these two studies focused on the short-course pretreatment setting of palbociclib as a chemoprotectant, whereas the present study is based on real-world safety data from its long-term antitumor treatment, reflecting the chronic effects of sustained CDK4/6 inhibition on gastrointestinal homeostasis. This time-effect divergence suggests a hypothesis that short-course pre-administration could be investigated for intestinal protection, whereas long-term treatment may warrant focused monitoring of the oral mucosa. Further research is needed to test this hypothesis.

Ribociclib generated the fewest positive PT-level disproportionality signals among the three CDK4/6 inhibitors. However, several signals were of notably high magnitude, including reflux gastritis (ROR = 13.65), defecation disorder (ROR = 11.38), and dysbiosis (ROR = 13.65). Severe lower GI AEs, such as intra-abdominal fluid collection and anal hemorrhage, were also identified through signal analysis. Notably, none of these significant positive disproportionality signals are currently documented in the prescribing information for ribociclib (5), which lists nausea, diarrhea, constipation, vomiting, stomatitis, and abdominal pain as common GI adverse reactions. One potential explanation for this discrepancy is the limited sample size and relatively short follow-up duration of clinical trials, which are insufficient to fully capture rare adverse events or those emerging after prolonged exposure. Moreover, stomatitis showed no significant reporting association with ribociclib in the present analysis. In the FAERS database analyzed in this study, ribociclib exhibited a distinctive GI adverse event reporting pattern, in which GI events represented only 10.58% of its total AE reports, but a high proportion were classified as serious (82.38%). However, because spontaneous reporting data lack exposure denominators and are subject to differential reporting biases, such as selective reporting of severe events, channeling of higher-risk patients, and regional variations in reporting practices, these proportions cannot be interpreted as evidence of lower incidence or higher inherent severity. These findings are preliminary and cannot directly inform clinical decision-making.

The reported GI AEs for abemaciclib were predominantly lower gastrointestinal events. Diarrhea was the most frequently reported GI event for abemaciclib and showed the strongest disproportionality signal (*n* = 2,790, ROR = 6.69), which aligns with prior clinical studies and prescribing information that identify diarrhea as a common GI adverse reaction and a leading cause of treatment interruption or dose reduction (6, 9, 24, 36). Several pharmacological properties of abemaciclib have been proposed in the literature as potential contributors to the diarrhea reported with this agent, although these mechanisms were not tested in the present study and require independent investigation. As one potential mechanistic hypothesis, abemaciclib displays approximately fivefold greater inhibitory potency for CDK4 relative to CDK6 (37), and the proliferation and homeostatic renewal of intestinal crypt epithelial cells are tightly dependent on CDK4 activity (38, 39). Preclinical animal studies have also demonstrated that abemaciclib inhibited glycogen synthase kinase 3β (GSK3β), with downstream activation of the Wnt/β-catenin pathway driving excessive proliferation of intestinal crypt cells, goblet cell depletion, and microvillus damage. These pathological changes are substantially less pronounced with palbociclib and ribociclib (40). Beyond its effects on GSK3β, abemaciclib has been reported to exhibit potent off-target inhibitory activity against CDK9, which may disrupt transcriptional regulation in intestinal epithelial cells, further exacerbating intestinal mucosal damage (28, 41). Additionally, abemaciclib markedly downregulated Ca^2+^/calmodulin-dependent protein kinase II (CaMKII), a key regulator of intestinal motility, further contributing to altered bowel function (42, 43). The continuous twice-daily dosing regimen of abemaciclib, which differs from the intermittent schedules of palbociclib and ribociclib, may also be a relevant factor, although its contribution to the observed reporting patterns cannot be determined from these data (4–6). Complementing this, we observed the highest reporting rate of monotherapy with abemaciclib (70.25%), a finding that raises the hypothesis that its adverse events may be driven primarily by its intrinsic pharmacological activity, rather than drug interactions. Beyond diarrhea, our analysis also identified significant disproportionality signals for abdominal pain upper (ROR = 4.13), hemorrhoidal hemorrhage (ROR = 3.93), and defecation urgency (ROR = 3.58), all of which are currently absent from its current prescribing information (6). The high reporting frequency and early onset of diarrhea observed in this database suggest that further prospective studies could evaluate proactive management strategies, including patient education on early symptom recognition. Unlabeled signals, such as swollen tongue and hemorrhoidal hemorrhage may also warrant assessment in such studies.

Based on the TTO analysis of 4,479 valid cases, heterogeneity in report-based temporal patterns across CDK4/6 inhibitors was observed. In this study, TTO was interpreted as the interval between the recorded therapy start date and event date as captured in FAERS, rather than as a biologically precise interval from drug exposure to adverse event onset. This heterogeneity must therefore be interpreted cautiously, as the recorded TTO values can be influenced by delays in diagnosis, reporting lags, and incomplete documentation. Analysis of the Weibull distribution shape parameter revealed that reported GI AEs of all CDK4/6 inhibitors in this database presented an early failure pattern (β < 1). However, recorded median TTO values varied significantly, with abemaciclib exhibiting the shortest onset (15.0 days), followed by ribociclib (27.0 days) and palbociclib (49.0 days). Abemaciclib accounted for 63.92% of all reported events within the first month of treatment, whereas 17.45% of reported palbociclib-associated GI AEs occurred beyond 1 year. This temporal distribution pattern may be associated with the dosing regimen of each agent. Abemaciclib is administered twice daily without interruption, which has been hypothesized to result in rapid attainment of steady-state plasma concentrations and the early reporting of GI events. In contrast, both palbociclib and ribociclib are given on an intermittent schedule of 3 weeks of treatment followed by 1 week off, which may provide a window for mucosal repair and potentially delay and disperse the peak reporting of GI events. Late-onset GI AEs of palbociclib are hypothesized to be less likely related to acute mucosal toxicity, but rather associated with long-term therapy-induced intestinal dysbiosis and cumulative immune-mediated reactions, although these speculations cannot be confirmed by the present pharmacovigilance analysis. With this caveat in mind, the observed temporal distinctions may inform hypotheses for agent-specific monitoring strategies. The first 4 weeks may represent a priority monitoring period for abemaciclib. Palbociclib may benefit from both early symptom surveillance and prolonged long-term follow-up. When patients present with new gastrointestinal complaints such as abdominal distension and abdominal pain after a stable treatment course, clinicians may consider a potential drug-related etiology to avoid misdiagnosis of disease progression or concurrent comorbidities. These monitoring strategies remain exploratory and require prospective validation.

Through a descriptive comparison of characteristics between serious and non-serious cases, this study identified several characteristics that were more frequently reported in serious GI AEs reports. These included advanced age, lower body weight, concomitant use of aromatase inhibitors or fulvestrant, and the presence of specific GI symptoms such as dyschezia, reflux gastritis and salivary hypersecretion. These characteristics represent features associated with serious reports in this dataset and should not be interpreted as independently validated risk factors, given the absence of multivariable adjustment and the inherent limitations of spontaneous reporting data. Ribociclib was disproportionately represented in serious GI AEs reports in this database, a finding consistent with its previously documented higher proportion of serious cardiovascular event reports among CDK4/6 inhibitors (44). However, these observations could not establish causal relationships or comparative risk profiles, and warrant further investigation in well-characterized cohorts capable of evaluating independent associations.

Notwithstanding the distinct GI adverse event reporting patterns observed among CDK4/6 inhibitors in this analysis, several inherent limitations of this spontaneous reporting system warrant careful consideration (15, 19). First and foremost, FAERS lacks reliable denominator data on total drug exposure, which precludes the calculation of incidence rates, absolute risks, or comparative risk estimates between different agents. Second, reporting biases inherent to spontaneous reporting systems, including systematic underreporting of mild to moderate adverse events, heightened clinical focus on severe reactions, and differential reporting related to time on market and channeling, may affect the observed reporting patterns. Third, the absence of critical clinical data, particularly regarding cancer staging and gastrointestinal history, restricts proper adjustment for potential confounders in the disproportionality analysis. Furthermore, the geographical concentration of reports from Western populations limits generalizability to Asian patients, whose distinct genetic backgrounds, clinical management paradigms, and underlying diseases may influence both drug susceptibility and the profile of reported events. Methodologically, disproportionality analysis can identify statistical associations but cannot establish exposure-response relationships, differentiate between acute and cumulative effects, or confirm causality. Moreover, for rare signals with high disproportionality, the limited number of cases may compromise stability and carry a risk of false positives. Thus, all reported patterns remain hypothesis-generating and do not constitute direct clinical guidance or comparative safety evidence.

## Conclusion

This pharmacovigilance study systematically characterized the heterogeneous disproportionate reporting patterns of GI AEs among CDK4/6 inhibitors in breast cancer patients using real-world data from FAERS. Abdominal discomfort emerged as a class-wide disproportionality signal. Palbociclib was disproportionately associated with oral mucosal events, abemaciclib showed a pronounced disproportionality for diarrhea and had the shortest median time to onset in reported cases, while ribociclib reports exhibited a higher proportion of serious outcomes along with infrequent but statistically elevated signals, such as reflux gastritis and dysbiosis. The majority of these PT-level signals were absent from current prescribing information. Time-to-onset analysis revealed differential temporal patterns that could inform hypotheses for agent-specific monitoring timeframes. Advanced age, low body weight, and concomitant aromatase inhibitors or fulvestrant were characteristics more frequently reported among serious cases, although these are not independently validated risk factors. These hypothesis-generating observations do not establish incidence, causality, or inter-drug risk differences but may help prioritize drug-event pairs for further validation in well-designed studies. Additionally, these data provide preliminary toxicological references for the potential use of CDK4/6 inhibitors in gastrointestinal malignancies, which also requires prospective confirmation.

## Data Availability

The datasets presented in this study can be found in online repositories. The names of the repository/repositories and accession number(s) can be found below: The datasets generated and/or analyzed in this study are publicly available from the FDA Adverse Event Reporting System database (https://fis.fda.gov/extensions/FPD-QDE-FAERS/FPD-QDE-FAERS.html), and data analyzed during the study are available from the corresponding author upon reasonable request.
