# Supplementary material for: Gastrointestinal toxicity associated with cyclin-dependent kinase 4/6 inhibitors in breast cancer patients: insights from a real-world pharmacovigilance analysis
Source: Front Med (Lausanne). 2026 Jul 15;13:1876415. doi: 10.3389/fmed.2026.1876415 (PMC13416550; doi:10.3389/fmed.2026.1876415)
Supplement: Supplementary file 1 [file Table_1.docx]

**Supplemental Table**

**Table Statistical formulas and thresholds for disproportionality analysis methods**

| Algorithm | Formula | Threshold |
| --- | --- | --- |
| ROR | ROR = $\frac{(a/c)}{(b/d)}$ = $\frac{ad}{bc}$  95%CI = $e^{ln(ROR)\pm1.96\sqrt{(\frac{1}{a}+\frac{1}{b}+\frac{1}{c}+\frac{1}{d})}}$ | a ≥ 3 and 95% CI lower limit for ROR > 1 |
| PRR | 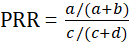PRR = $\frac{a/(a+b)}{c/(c+d)}$  $\chi^{2}$ = $\frac{{(ad-bc)}^{2}(a+b+c+d)}{(a+b)(a+c)(c+d)(b+d)}$ | a ≥ 3, PRR ≥ 2 and χ² ≥ 4 |
| BCPNN | IC=$\log_{2} \frac{a(a+b+c+d)}{(a+c)(a+b)}$  IC025=E(IC)-2[V(IC)]^0.5^ | IC025 > 0 |

a, number of target event reports of target drugs; b, other event reports of the target drug; c, target event reports of other drugs; d: other event reports of other drugs; ROR, reporting odds ratio; PRR, proportional reporting ratio; BCPNN, Bayesian Confidence Propagation Neural Network; CI, confidence interval; IC, Information component; IC025, 95% CI lower limit for IC; E (IC), IC expectation; V (IC), IC variance.
